# Neonicotinoid-Coated *Zea mays* Seeds Indirectly Affect Honeybee Performance and Pathogen Susceptibility in Field Trials

**DOI:** 10.1371/journal.pone.0125790

**Published:** 2015-05-18

**Authors:** Mohamed Alburaki, Sébastien Boutin, Pierre-Luc Mercier, Yves Loublier, Madeleine Chagnon, Nicolas Derome

**Affiliations:** 1 Université Laval, Institut de Biologie Intégrative et des Systèmes (IBIS), Québec, Canada; 2 Université Laval, Département de biologie, Faculté des sciences et de génie, Québec, Canada; 3 Centre de Recherche en Sciences Animales de Deschambault (CRSAD), Québec, Canada; 4 Université du Québec à Montréal, Québec, Canada; 5 CNRS, Laboratoire Evolution, Génomes et Spéciation LEGS, Gif-sur-Yvette, France; Rutgers University, UNITED STATES

## Abstract

Thirty-two honeybee (*Apis mellifera*) colonies were studied in order to detect and measure potential *in vivo* effects of neonicotinoid pesticides used in cornfields (*Zea mays* spp) on honeybee health. Honeybee colonies were randomly split on four different agricultural cornfield areas located near Quebec City, Canada. Two locations contained cornfields treated with a seed-coated systemic neonicotinoid insecticide while the two others were organic cornfields used as control treatments. Hives were extensively monitored for their performance and health traits over a period of two years. Honeybee viruses (brood queen cell virus BQCV, deformed wing virus DWV, and Israeli acute paralysis virus IAPV) and the brain specific expression of a biomarker of host physiological stress, the Acetylcholinesterase gene AChE, were investigated using RT-qPCR. Liquid chromatography-mass spectrometry (LC-MS) was performed to detect pesticide residues in adult bees, honey, pollen, and corn flowers collected from the studied hives in each location. In addition, general hive conditions were assessed by monitoring colony weight and brood development. Neonicotinoids were only identified in corn flowers at low concentrations. However, honeybee colonies located in neonicotinoid treated cornfields expressed significantly higher pathogen infection than those located in untreated cornfields. AChE levels showed elevated levels among honeybees that collected corn pollen from treated fields. Positive correlations were recorded between pathogens and the treated locations. Our data suggests that neonicotinoids indirectly weaken honeybee health by inducing physiological stress and increasing pathogen loads.

## Introduction

Honeybee populations around the world have declined significantly in the last decade [1, 2]. The phenomenon of global honeybee decline represents a major challenge for beekeepers and scientists alike. Its causes are still not well understood. Several studies highlight the impact of endemic and emergent pathogens [3–7]; others blame the excessive use of pesticides [1, 8]. Multiple chemical residues of synthetic origin have been detected inside honeybee hives, including pesticides used in varroa treatment [9, 10]. However, no individual factor such as environment, pesticide or pathogen, seems to act as the principal driver of Colony Collapse Disorder (CCD) or other honeybee losses. Thus the massive decline of honeybee populations in the world is widely considered a multifactorial phenomenon [11].

The decline of bee populations has significant implications for plant pollination, including many domesticated crops [12]. Indeed, several authors envision a looming pollination crisis that will threaten worldwide food security [13, 14]. The value of crops pollinated by bees was estimated in 2000 at $14.6 billion US dollars in the United States alone [15]. In the United States, total honeybee colony number has declined by 45% over the past 60 years [16]. The majority of pre-1979 losses were attributed to organochlorine, carbamate and pyrethroid pesticide exposure [17]. In Canada, colony losses seem to be less severe than in the USA, although the data is less complete. Wintering losses in 2009–2010 were reported at 23.8% [18] while other studies show a decrease of honeybee mortality in Canada from 35% in 2007 to 15% in 2012 [19].

Over the years, the classes of pesticide used in agriculture and their application methods have shifted substantially. Carbamates, pyrethroids and organochlorides, known for their environmental toxicity and traditionally sprayed directly onto crop plants, have been less used for the favor of new classes of systemic pesticides (neonicotinoids and phenylpyrazoles). Neonicotinoids and phenylpyrazoles, commonly applied as seed-coatings to limit contact with non-target plants and insects, were thought to be less harmful for pollinators. However, various *in vitro* studies have revealed the high toxicity of neonicotinoids such as clothianidin and thiamethoxam to the honeybee [20]. In the field, lethal pesticide toxicity among honeybees has been widely studied across multiple classes of synthetic agents [8, 18, 21–24]. In such cases, lethal toxicity is easily confirmed via the presence of dead bees in front of the hives. However, fewer studies deal with the effects of sublethal doses of pesticides on honeybees. It is known that the sublethal doses deplete the essential activities of insects [25–28] even at concentrations below the detection limits of analytical chemistry [29]. Sublethal doses significantly decrease honeybee performance and trigger disorders in colony dynamics and labor partition [24, 30]. Moreover, it has been proved that honeybee behavior, orientation, communication dances and return flights, especially for foragers, are highly affected by sublethal pesticide doses [31, 32]. Sublethal doses of neonicotinoids in particular are known to impair the olfactory memory and learning capacity of honeybees [33–35] and mar the flying behavior and navigational capacity of bee foragers [36, 37]. Currently available analytic chemistry methods, such as liquid chromatography-mass spectrometry techniques, have a very low limit of detection LOD for neonicotinoids [38]. However, infield assessment of both neonicotinoid sublethal exposure and its consequent toxic effect needs the development of integrative tools, which combine both highly sensitive physiological biomarkers and chemical detection techniques. Because data concerning insecticide-induced behavioral perturbations is necessarily quantitative in nature, we targeted the efficiency of a quantitative biomarker of neurophysiological stress.

A recent study has linked neonicotinoid sublethal toxicity with an increase in Acetylcholinesterase (AChE) activity in the honeybee [39]. Expressions levels of this neuromodulator thus provide a valuable quantitative proxy. Therefore, expression levels of this new biomarker were targeted in this study together with more classical measures of hive condition, in order to assess any potential effects of neonicotinoid-coated *Zea mays* spp (henceforth ‘corn’) seeds on honeybee health. In order to more faithfully reflect the nature of the *in vivo* neonicotinoid impacts on honeybee health, we investigated both neonicotinoid toxicity and any potential synergy linking the proximity of neonicotinoid treated cornfield with the studied pathogens and the AChE expression. Multiple longitudinal comparisons between colonies were made in the context of natural bee foraging activity and in an experimental system comprising of replicated treated and untreated cornfields in order to isolate the treatment effect. Our results show that honeybee colonies foraging near treated cornfields demonstrated significantly higher AChE expression, increased viral loads as well as increased varroa infestation during the year of study. Aside of these significant results, classic measures of hive condition—mass and brood count—exhibited fewer disturbances.

## Materials and Methods

### Ethics statement

No specific permission was required to run this study in these locations. Our field studies did not involve endangered or protected species. The GPS coordinates for each location were as follows: N°1 (46°40’31 N 71°54’57 W), N°2 (46°38’38 N 71°56’56 W), N°3 (46°40’04 N 72°00’26 W) and N°4 (46°35’04 N 72°14’58 W).

### Honeybee colonies and locations

**Fig 1:**
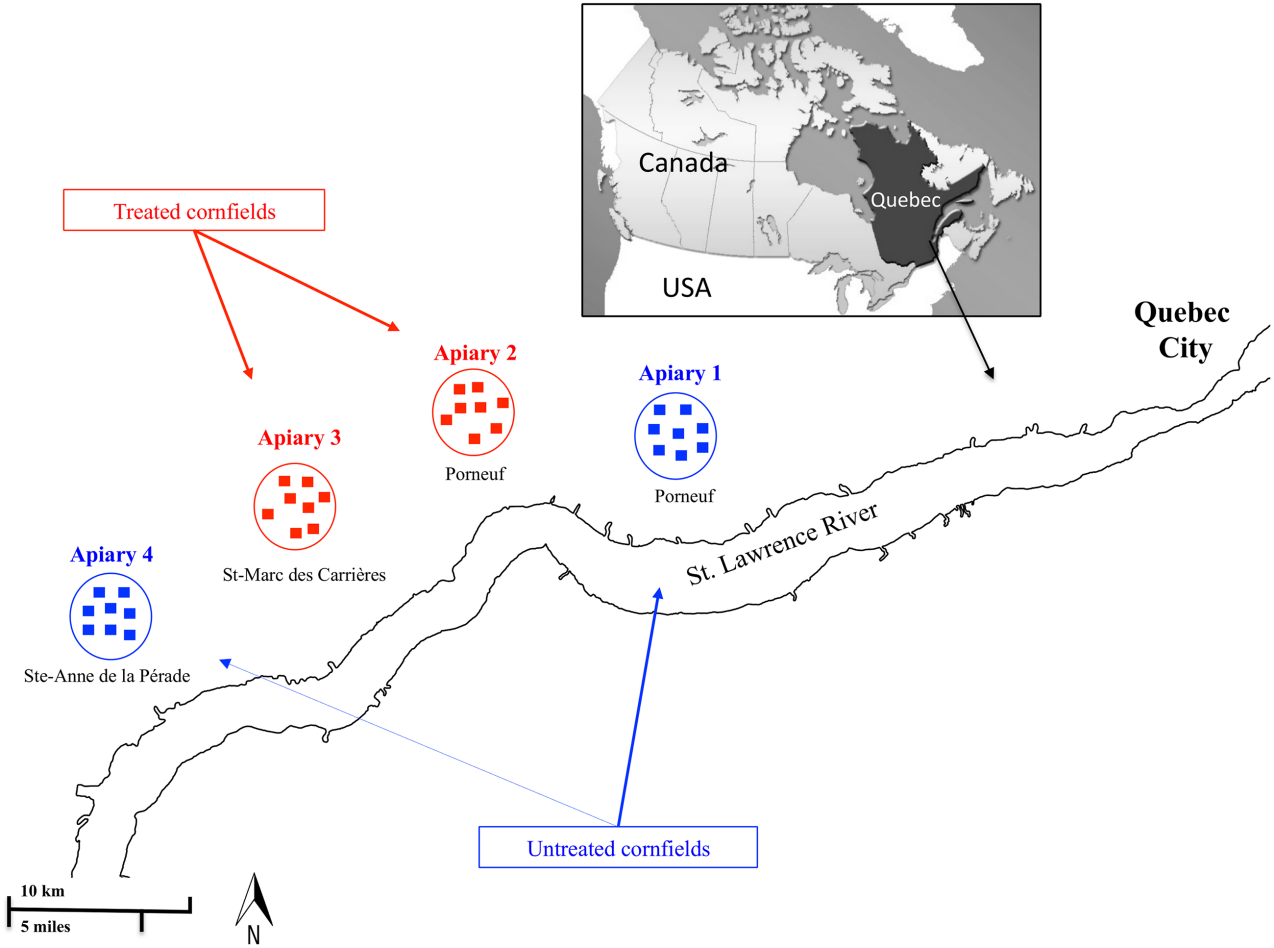
Location of the four honeybee apiaries southwest of Quebec City. Each apiary consisted of eight honeybee hives. Apiaries 1 and 4 (bleu color) are located in untreated cornfields while apiaries 2 and 3 (red color) are in fields sown with neonicotinoid treated seeds. Each square represents one honeybee hive.

This study was based on 32 managed honeybee colonies, provided by a local beekeeper in Quebec. Colonies were all new healthy divisions of 2012, equal in population size, provided with newly fertilized and tested queens. Honeybees were received on 28-June 2012 in temporary hives. Then they were moved to 32 new Langestroth hives. Colonies were split into four apiaries of 8 colonies each on 1-July 2012. Apiaries were distributed in four different clusters of cornfields southwest of Quebec (Fig 1 and Table 1).

**Table 1:** Timetable of all the procedures taken in this study.

	Apiary 1	Apiary 2	Apiary 3	Apiary 4
**Geographical location**	Portneuf	Portneuf	St-Marc des Carrières	Ste-Anne de la Pérade
**Number of colony**	8	8	8	8
**Colony placed in field**	1-July	1-July	1-July	1-July
**Cornfield treatment**	Untreated	Treated	Treated	Untreated
**Treatment applied**	None	Seed-coating	Seed-coating	None
**Pesticide applied**	None	Neonicotinoid insecticide (Cruiser)	Neonicotinoid insecticide (Cruiser)	None
**Active molecule**	None	Thiamethoxam and or Clothianidin	Thiamethoxam and or Clothianidin	None
**Corn flowering**	5- August			
**Sampling corn flower**	9-August			
**Sampling N°1 (50 + 100 honeybees/colony)**	13-July			
**Sampling N°2 (50 + 100 honeybee/colony)**	23-August			
**Sampling N°3 (50 + 100 honeybee/colony)**	2-September			
**Sampling N°4 (50 + 100 honeybee/colony)**	15-January			
**Pollen sampling N°1**	2-August			
**Pollen sampling N°2**	9-August			
**Pollen sampling N°3**	23-August			
**Pollen sampling N°4**	6-September			
**Honey sampling (100 ml/colony)**	20-September			
**Brood photography**	30-July, 16-August, 31-August and 13-September			
**Varroa counting**	9-August, 23-August, 2-September and 15-September			
**Colony weight recording (2012–2013)**	20-August 2012 to 10-April 2013			

Sampling dates of honeybees, pollen and seeds samples are mentioned as well as the pesticide and the active molecules used in each field. Dates of varroa mite counting, brood photography and colony weight recordings for all apiaries are also provided.

Two apiaries (N°1 and 4) were placed in agricultural cornfield areas that did not use seed treatment, while apiaries (N°2 and 3) were placed in cornfields treated via seed-coating with a commercial insecticide with thiamethoxam as an active ingredient (Cruiser, Syngenta, Canada) belonging to the neonicotinoid class, Table 1. The four apiary sites are located in a geographical area where climate, environmental conditions and flora are very similar. Apiary sites (N°1 and 4) are located in two organic cornfield areas while apiary sites (N°2 and 3) mainly contain huge cornfields treated with neonicotinoid coated-seeds. Under normal conditions, honeybee foragers fly between (1.5–5) km distance if abundant sources of food are nearby [40]. In our case, the shortest distance was 5 km, between both treated fields (N°2 and 3). This assumes that a cross contamination is far to occur, as the shortest distance between studied locations was about 5 km. However, both other apiaries (untreated locations) were well isolated as the distance was more than 20 km between both (N°2 and 3) and N°4 (Fig 1). To our knowledge, no other chemical pesticides were used on the treated cornfields such as fungicides or herbicides. We do not exclude the probable use of such pesticides on other cultures in the treated areas. Crop rotations in the four studied areas (N°1, 2, 3 and 4) are most of the time concentrated between corn *Z*. *mays* and white clover *Trifolium* sp.

### Adult bee and honey sampling

Fifty adult worker bees per colony were sampled four times; before, during and after corn flowering, as well as one time point during the wintering (Table 1). Honeybees were flash frozen in liquid nitrogen for 10 seconds and immediately put on dry ice until arriving to the lab and stored at -80°C. Among the fifty bee samples of each colony, twenty-five bees were randomly picked and used for further molecular studies. Samples left over were kept as a backup for further analysis. In addition, another 100 worker bees were sampled from each colony in the same four time points mentioned above. These hundred bees coming from each of the eight apiary’s colonies, were pooled and treated as one sample per apiary for each time point and were used for pesticide chemical detection. Honey samples were collected from each hive at the end of the corn flowering period. Multiple honey samples were taken from different honey frames of each hive. Honey samples collected from the eight hives of each location were pooled and treated as one sample per apiary in subsequent analyses (Table 1). In addition, corn flowers from each site were randomly sampled during the flowering period and conserved at -20°C for chemical analysis.

### MtDNA analysis

In order to determine the maternal origin of the studied colonies, a COI-COII mtDNA test [41–44] was performed on worker bees of each colony. Briefly, DNA was extracted from the thorax using the Chelex method [45], standard PCR amplification of the COI-COII intergenic region of the mtDNA was performed followed by an electrophoresis migration on 1.4% agarose gel with the molecular marker MIII (Sigma-Aldrich Biotechnology) and amplified fragment size variation used to determine lineage. The evolutionary lineage of each studied colony was determined according to the different patterns of the amplified mtDNA COI-COII intergenic region (ex. Q pattern for the North Mediterranean lineage C and (PQ, PQQ, PQQQ) patterns for the West Mediterranean lineage M) [41–44].

### RNA extractions

Total RNA was extracted from honeybee brain and abdomen using TRIzol Reagent protocol from Invitrogen [46] with some modifications. Briefly, the brains and abdomens of 25 bees from each colony were dissected and added separately to 1 mL Trizol with 5 mg of acid washed glass beads and gently mixed for 2 min. 300 μL of chloroform was added and the total mixture was incubated at room temperature for 15 min followed by a centrifugation at 12000 rpm for 15 min at 4°C. The supernatant was then washed with 250 μL each of isopropanol and 1.2 M sodium citrate with incubation for 10 min at room temperature, followed by centrifugation at 12000 rpm for 10 min at 24°C. The pellet was subsequently washed twice with 1 mL 75% ethanol and centrifuged at 12000 rpm for 10 min at 24°C. Finally, the RNA pellet was dried and 30 μL of nuclease-free water was added. RNA was stored at -80°C for further analyses.

### AChE gene expression and pathogens detection

One-step reverse transcription quantitative PCR (RT-qPCR) was used to quantify the expression of the Acetylcholinesterase gene (AChE), as well as to evaluate the viral load for three of the most common viruses in Canada: 1- Black queen cell virus (BQCV), 2- Deformed wing virus (DWV) [47] and 3- Israeli acute paralysis virus (IAPV) [48]. Positive and negative controls for each virus were generated from current stocks and run in every RT-qPCR. Glyceraldehyde-3-phosphate dehydrogenase (GAPDH) and Ribosomal protein S18 (RPS18) genes were used as reference genes for AChE expression and virus detection respectively in all RT-qPCR [49]. Reference genes were selected after many tests performed based on their accurate results and stability on inter and intra bee tissues [49]. QScript One-Step SYBR Green RT-qPCR kit from Quanta—Bioscience was used to perform all qPCR analyses for both AChE expression and viral detection. The standard protocol of Quanta kit for all the RT-qPCR was applied using 2 μL of 0.1 μg purified RNA.

### Varroa mite infestation

Each studied colony had been equipped with a sticky bottom board for varroa mite count. Passive varroa mite counts were made from this board four times for each colony during the period of peak activity (August-September), which coincides with corn flowering. Sticky bottom board counts were left 72h and were used to estimate differences in varroa abundance between colonies located in treated and untreated cornfields, as well longitudinally across all hives. No chemical treatments for varroa were applied during the experiment.

### Pollen collection and analysis

Pollen was collected from hives of each apiary using pollen collectors fixed in the hives’ entrances. Pollen was collected at different times: before (2-August-12), during (9-August-12) and after the corn flowering period (23-August and 6-September, 2012), (Table 1). Pollen collected from the hives of each location were pooled, desiccated at 37°C for 48h and conserved at -20°C. Each dry sample was very well mixed and 10 g of each sample was randomly sampled for pollen determination. For each 1 g sample, the botanical origin of pollen loads was determined with 2000 to 4500 observed pollen grains. The taxonomic diversity of pollen samples for each sampled date and locality was determined by observing the total surface of slides [50].

### Pesticide detection

The presence of more than 150 pesticides was evaluated in worker bees in this study, as well as in pollen, corn flowers and honey using Liquid chromatography-mass spectrometry (LC-MS) method [38]. The limit of detection LOD for neonicotinoids is 0.6 μg/kg for a limit of quantification LOQ of 2 μg/kg [38]. Analyses were processed at the laboratory of the Ministère de l'Agriculture, des Pêcheries et de l'Alimentation du Québec (MAPAQ). Pollen samples collected from hives of each apiary were pooled after thorough homogenization. In total, 16 pollen samples were analysed for pesticide residues as well as 12 honeybee workers, 4 honey and corn flower samples. Five grams of each pollen, honeybee workers (50 bees) and honey (2.5–3.0 ml of liquid honey) samples were used for pesticide detection.

### Hive condition

Two main parameters were evaluated to measure the biological development of each experimental hive: weight (kg) and the colony brood development [51, 52]. A weekly record was kept of the hive’s weight during summer (March-June), spring (June-September) and fall seasons (September-December) of 2012. During indoor wintering, two measurements were taken (15-January and 26-March, 2013). To evaluate brood development, all bee frames containing capped brood cells were photographed twice per month during the period of activity. Surface area estimations of brood frequency are deemed to be insufficiently accurate, thus capped worker brood cells were counted by manual dotting using Image J software [53]. The total capped worker cells counted for each colony reflects the exact number of eggs laid by the queen in a given time of the brood cycle.

### Statistical analysis

All statistical analyses for the AChE gene expression, viruses prevalence (DWV, BQCV and IAPV), varroa mite load, brood development and hive weight for all the colonies were performed using linear mixed-effects models [54]. These models are provided in the “lme4” package [55] for maximum likelihood or restricted maximum likelihood (REML) parameters estimation and the “LmerTest” [56] package in order to perform likelihood ratio test (LRT) and F-tests for random and fixed factors. Statistical analyses were performed on sixteen independent biological replicates for each treatment and each date. Those sixteen replicates were technically replicated three times for each RT-qPCR quantification. Statistical analyses concerning AChE expression of colonies that have collected corn pollen were performed on five colonies (two located in treated and three in untreated locations) which were biologically replicated five time for each colony.

Each studied variable (AChE expression, DVW, BQCV and IAPV prevalence, varroa load, brood and weight) was tested for normality with the Shapiro-Wilk test [57]. Variables not normally distributed were normalized for their distribution by log transformation. In the linear mixed models used in our statistical analyses, the factor ‘apiary location’ was always considered as a random factor in order to assess any potential effect of the different apiary locations. Sampling time point was treated as a ‘repeated measure’ and when overtime variables were analyzed, the factor ‘date’ was treated as a fixed effect. The linear mixed models were fit by maximum likelihood and the Welch-Satterthwaite t-test was used [58].

Correlations between the studied variables and the treatment factor were tested using the same models described above on overtime observations and by allowing interaction between variable. In the linear mixed models used to generate the correlation matrixes, date was considered a fixed factor as overtime data were tested and apiary location as a random factor to fairly evaluate the treatment’s effect in the dataset. All statistical analyses were carried out in the R environment [59].

## Results

### Colonies genetic background

All the studied colonies have shown a (Q) pattern for their mtDNA COI-COII intergenic region. Thus, they all belong to the North Mediterranean lineage (C).

### Pollen analysis

Palynological analyses for each apiary on the four different sampled dates (2, 9, 23-August and 6-September, 2012) (Table 1) revealed various types of pollen. *Trifolium* sp. (Fabaceae) was the most visited flower (> 45%) followed by *Lythrum* sp. (Lythraceae) at 12–45%. Several species of *Solidago* (Asteraceae) were also recorded at 3–15%. Corn pollen *Z*. *mays* (Poaceae) was identified in five hives (R2, R8, R12, R24 and R26) at an abundance of c. 1% of total (S1 Fig).

### AChE expression

**Fig 2:**
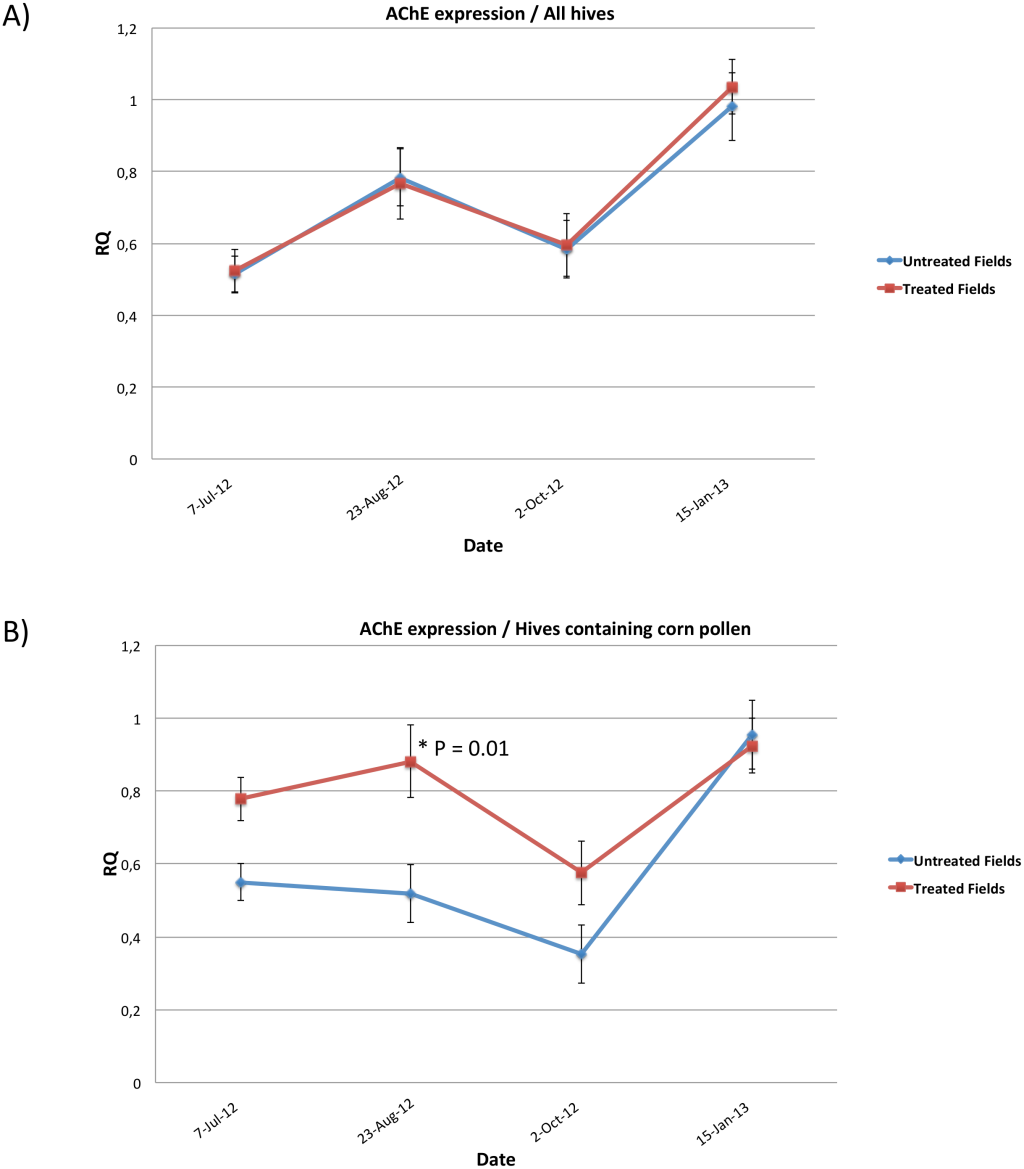
a) Means of Acetylcholinesterase (AChE) expression on four dates for all studied hives, located in treated and untreated fields b) Means of AChE expression on four dates for colonies that had collected corn pollen: (R12 and R24) in treated and (R2, R8 and R26) in untreated cornfields. RQ is the relative quantity of the virus infection in the original samples, and error bars are the Standard Errors (SE) of each studied group. P value is * P < 0.05.

AChE expression levels for all studied samples are summarized in Table 2. The **ΔΔ C**_**T**_ mean was calculated, as well as the relative quantity of original template (RQ), for the colonies located in treated and untreated fields separately. P-value shows no significant difference for AChE expressions between colonies located in treated and untreated cornfields in the four sampled dates (Table 2, Fig 2a). However, when comparing AChE levels only for colonies that have collected corn pollen, significantly greater AChE expression (T = 2.62, P = 0.01) was observed on 23-August-12 as well as overtime expression (T = 2.22, P = 0.02) for colonies located in treated cornfields compared to those in untreated ones (Table 2, Fig 2b). Finally, in both treated and untreated fields, AChE expression levels varied significantly between sampling dates (T = 4.49, P < 0.001).

**Table 2:** Contrasts in honeybee pathogen abundance (deformed wing virus DWV, black queen cell virus BQCV and varroa) and AChE expression by date and neonicotinoid treatment.

	Neonicotinoid treated fields (Mean /16 colonies)			Untreated fields (Mean /16 colonies)			Treatment	
Target	ΔΔ C_T_	SE	RQ	ΔΔ C_T_	SE	RQ	T-value	P-value
DWV^(1)^	+5.45	0.14	0.042	+4.56	0.19	20.07	-0.85	0.44
BQCV^(1)^	+11.89	0.15	0.073	+10.83	0.17	0.28	-0.43	0.68
AChE^(1)^	+1.15	0.18	0.54	+1.03	0.23	0.53	-0.36	0.71
AChE^(1)^/ Corn Pollen only	+1.21	0.10	0.78	+1.06	0.11	0.56	0.97	0.34
Varroa^(1)^	-	-	-	-	-	-	1.18	0.30
DWV^(2)^	+3.99	0.24	0.35	+3.94	0.13	95.57	-0.08	0.92
BQCV^(2)^	+7.66	0.15	0.84	+12.75	0.16	0.04	2.85	0.007 **
AChE^(2)^	+0.63	0.17	0.80	+0.67	0.14	0.73	-0.26	0.79
AChE^(2)^/ Corn Pollen only	+1.10	0.19	0.90	+1.32	0.17	0.51	2.62	0.018 *
Varroa^(2)^	-	-	-	-	-	-	1.75	0.08
DWV^(3)^	+3.14	0.15	61.59	-0.05	0.15	406.11	-1.47	0.15
BQCV^(3)^	+7.46	0.13	0.13	+12.35	0.25	0	3.13	0.003 **
AChE^(3)^	+0.86	0.13	0.62	+0.87	0.12	0.61	0.04	0.96
AChE^(3)^/ Corn Pollen only	+0.98	0.18	0.59	+0.84	0.16	0.37	1.76	0.09
Varroa^(3)^	-	-	-	-	-	-	2.24	0.031*
DWV^(4)^	-2.78	0.18	2548.16	-7.56	0.15	5388.73	-1.43	0.22
BQCV^(4)^	+14.46	0.17	0.002	+18.20	0.26	0	2.01	0.11
AChE^(4)^	-0.14	0.12	1.14	+0.10	0.11	1.04	0.49	0.64
AChE^(4)^/ Corn Pollen only	+0.34	0.15	0.95	+0.23	0.17	0.97	-0.60	0.55
Varroa^(4)^	-	-	-	-	-	-	0.85	0.39
**Overtime**								
DWV							-1.34	0.24
BQCV							2.01	0.11
AChE							-0.08	0.93
AChE/ Corn Pollen only							2.22	0.029 *
Varroa							2.81	0.0056 **

^(1)^, ^(2)^, ^(3)^ and ^(4)^ are the sampled dates 13-July-12, 23-August-12, 02-October-12 and 15-January-13 respectively.

**ΔΔ C**_**T**_ is the threshold cycle in qPCR reactions, SE: the standard errors of **ΔΔ C**_**T**_ and RQ is the relative quantity of the RNA template in the original samples. P-value is the probability of RQ mean value by the Welch-Satterthwaite t-test on linear mixed models between colonies located in neonicotinoid treated and untreated cornfields. (-) means not applicable.

### Virus infection

**Fig 3:**
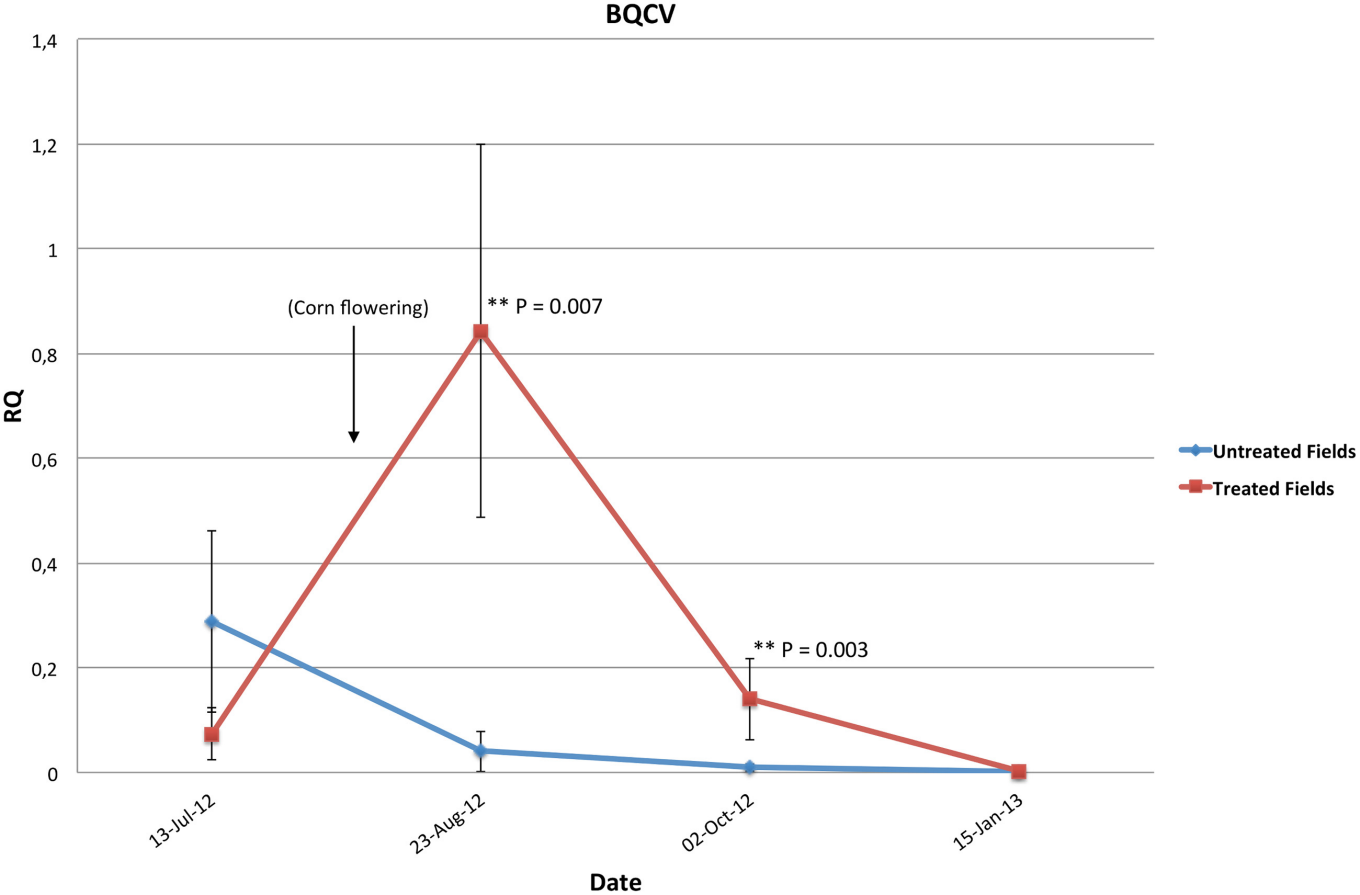
Mean level of the black queen cell virus (BQCV) infection for the 32 studied colonies, 16 colonies in each treated and untreated fields on four different dates. RQ is the relative quantity of the virus infection in the original samples. Error bars are the Standard Errors (SE) of each studied group. P values is ** P < 0.01.

RT-qPCR investigations for three viruses (BQCV, DWV and IAPV) revealed no IAPV in analyzed samples. However, BQCV and DWV have been identified at different dates. The highest mean level of BQCV infection was recorded at the end of the corn flowering period (23-August-12) for the colonies located in treated fields. Furthermore BQCV infection levels were significantly higher in colonies located in treated cornfields (T = 2.85, P = 0.007 and T = 3.13, P = 0.003) than in colonies of the untreated cornfields for dates 2 and 3 respectively (Table 2, Fig 3). DWV demonstrated a different pattern of infection prevalence to BQCV and peaked during winter (15-January-13) for both treated and untreated colonies (Table 2). No significant differences between colonies placed in treated and untreated cornfields were observed for DWV (T = -1.34, P = 0.24) (Table 2).

### Varroa mite abundance

**Fig 4:**
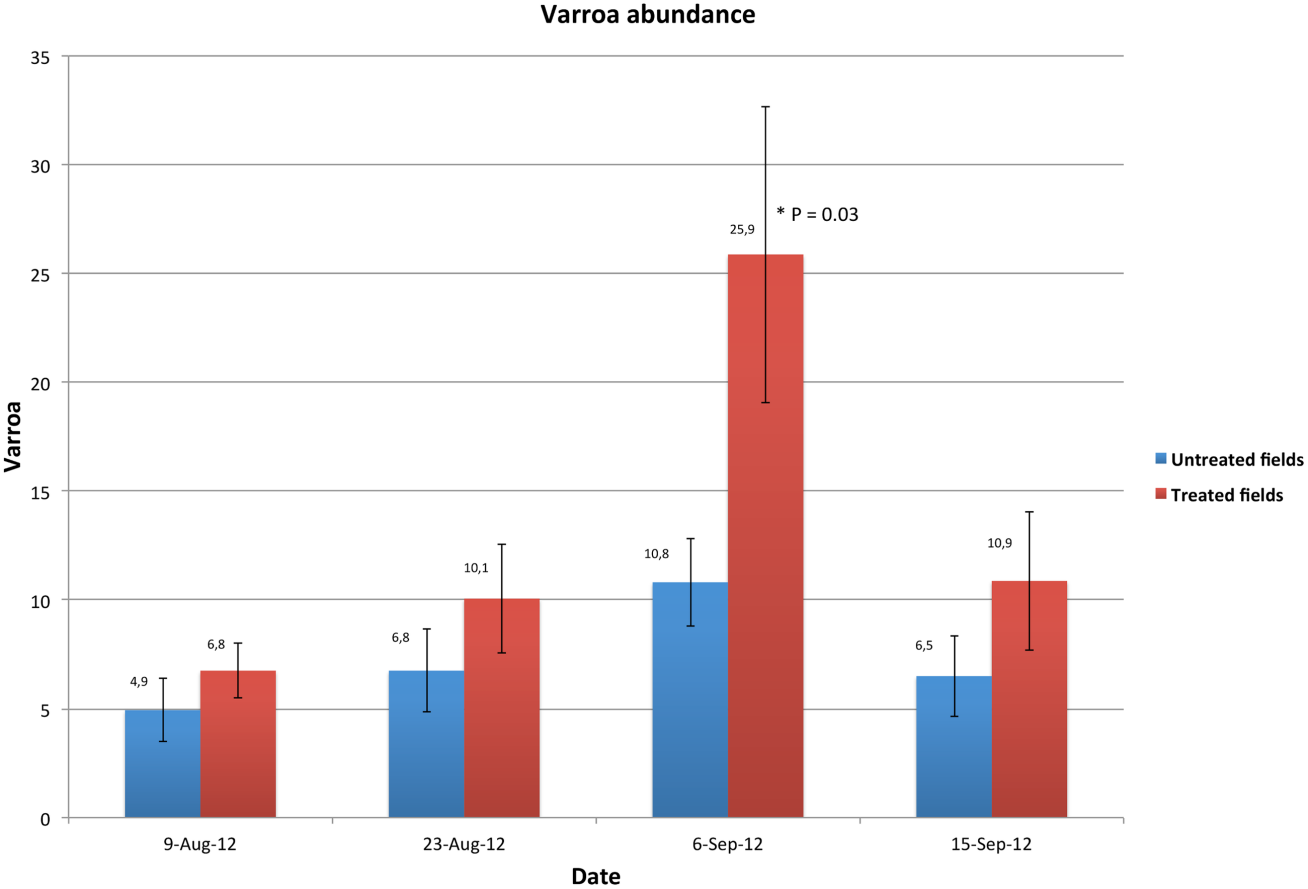
Mean values of varroa mite abundance in the 32 studied colonies, 16 colonies in each treated and untreated cornfields on four different dates. Error bars are the Standard Errors (SE) of each studied group. P values is * P < 0.05.

Varroa infestation was higher in hives located in treated cornfields on all studied dates (Fig 4). The highest mean of counted varroa mites was observed in 6-September-12 in the colonies of the treated cornfields with a significant P-value (T = 2.24, P = 0.031) (Table 2, Fig 4). Throughout the dates, varroa load was highly significant in colonies of the treated cornfields compared to those of the untreated ones (T = 2.81, P = 0.005) (Table 2).

### Chemical analyses

Detectable pesticide residues on pollen, adult bees, honey and corn flowers for each date are summarized in Table 3. Neonicotinoid pesticides were not detected in honey sampled from the four apiaries in 20-Sep-12. Thiabendazole, a fungicide, was detected in all honey samples at very low concentrations (0.0004–0.0008 μg/g). Among adult honeybees, no pesticides were detected in any samples at any time points, except for the samples coming from apiary 3 on 13-July-12, in which low levels of Atrazine (herbicide) were detected. Very low levels of carbaryl (insecticide) were identified in some pollen samples. Thiamethoxam was not detected while clothianidin—a neonicotinoid pesticide—which is a metabolite of thiamethoxam, was identified in the corn flowers of the cornfields N°3 at low concentration (0.0037 μg/g) (Table 3, Fig 1).

**Table 3:** Chemical pesticide residue analyzed by (LC-MS) for honey, adult bee, pollen and corn flower from the four studied locations on different dates.

	Apiary 1 μg/g	Apiary 2 μg/g	Apiary 3 μg/g	Apiary 4 μg/g	Pesticides	Type	LD_50_ (Adult bee) μg/g
**Honey**	0.0008	0.0004	0.0008	0.0008	Thiabendazole	Fungicide	> 2000
20-Sep-12							
**Adult bee**	-	-	0.022	-	Atrazine	Herbicide	1113
13-Jul-12							
**Adult bee**	-	-	-	-	-	-	-
23-Aug-12							
**Adult bee**	-	-	-	-	-	-	-
02-Nov-12							
**Pollen**	0.0008	0.0026	0.0016	0.0006	Carbaryl	Insecticide	2
02-Aug-12							
**Pollen**	-	-	0.0008	-	Carbaryl	Insecticide	2
09-Aug-12							
**Pollen**	-	-	-	-	-	-	-
23-Aug-12							
**Pollen**	0.0062	-	-	-	Carbaryl	Insecticide	2
06-Sep-12							
**Corn flower**	-	-	0.0037	-	Clothianidin	Neonicotinoid	0.0037 μg/bee (Acute oral toxicity)
09-Aug-12							

(-) means chemical compound not found or below the level of detection (LOD). LD_50_ is based on the data provided by [60].

### Weight and brood developments

**Fig 5:**
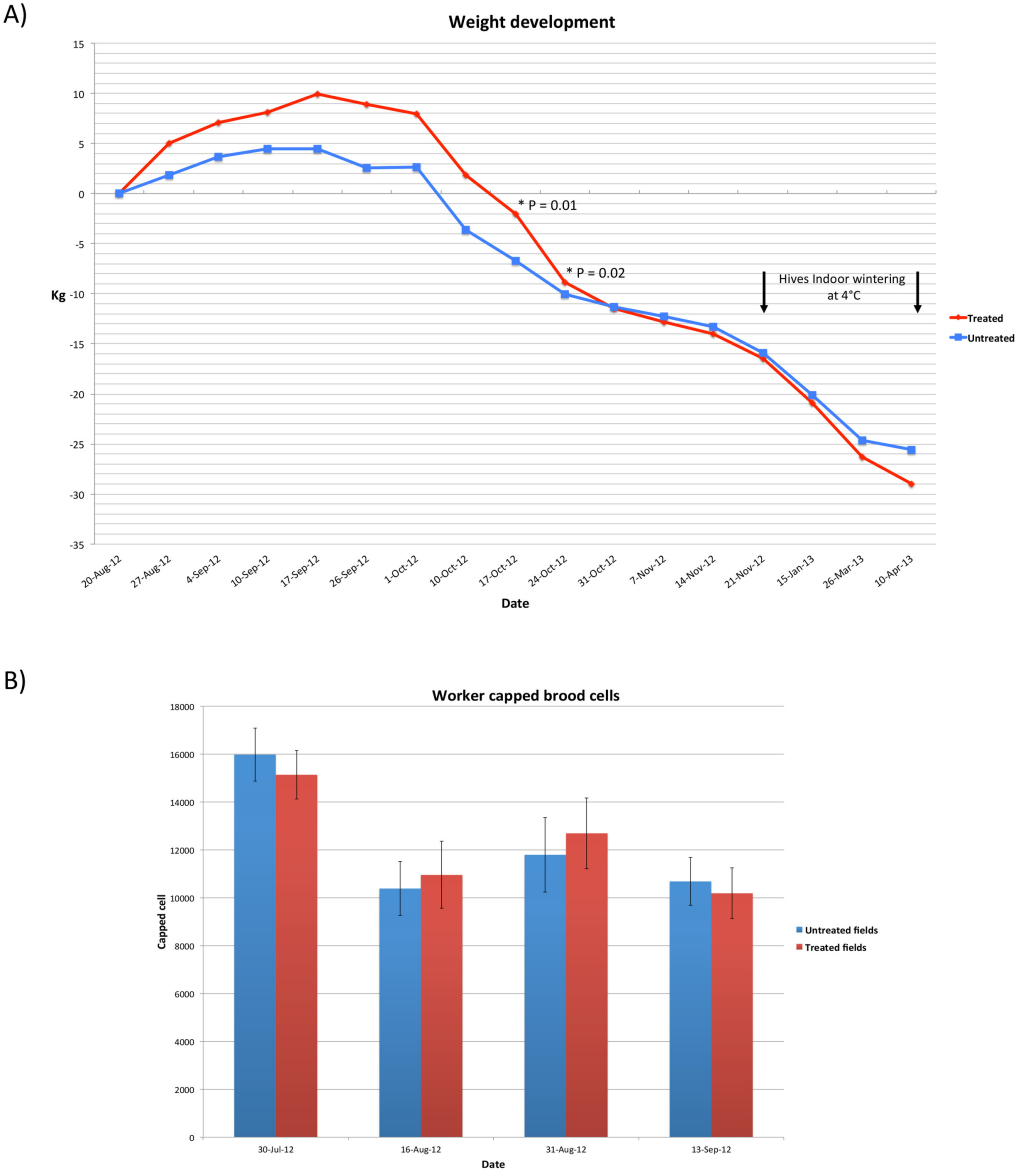
a) Differential weight of the hives mean weight values in treated and untreated fields b) Worker brood mean values of the two studied groups (16 colonies in each treated and untreated cornfields). Error bars are the Standard Errors (SE) of each group.

Differentials mean values were calculated for both colony groups: located in treated and untreated cornfields (Fig 5a). The total weight development was significantly higher (T = 2.48, P = 0.01 and T = 2.36, P = 0.02) in the colonies of the treated cornfields on two dates (17, 24-October-12) respectively (Fig 5a). Both groups showed equal weights during the wintertime from 31-October-12 until 15-January-13. From 26-March-13 onwards, the untreated group attained a greater mean weight than the treated group (Fig 5a). Brood development showed no significant difference between both treated and untreated groups in the four studied dates (Fig 5b).

### Pathogen—treatment correlation

**Fig 6:**
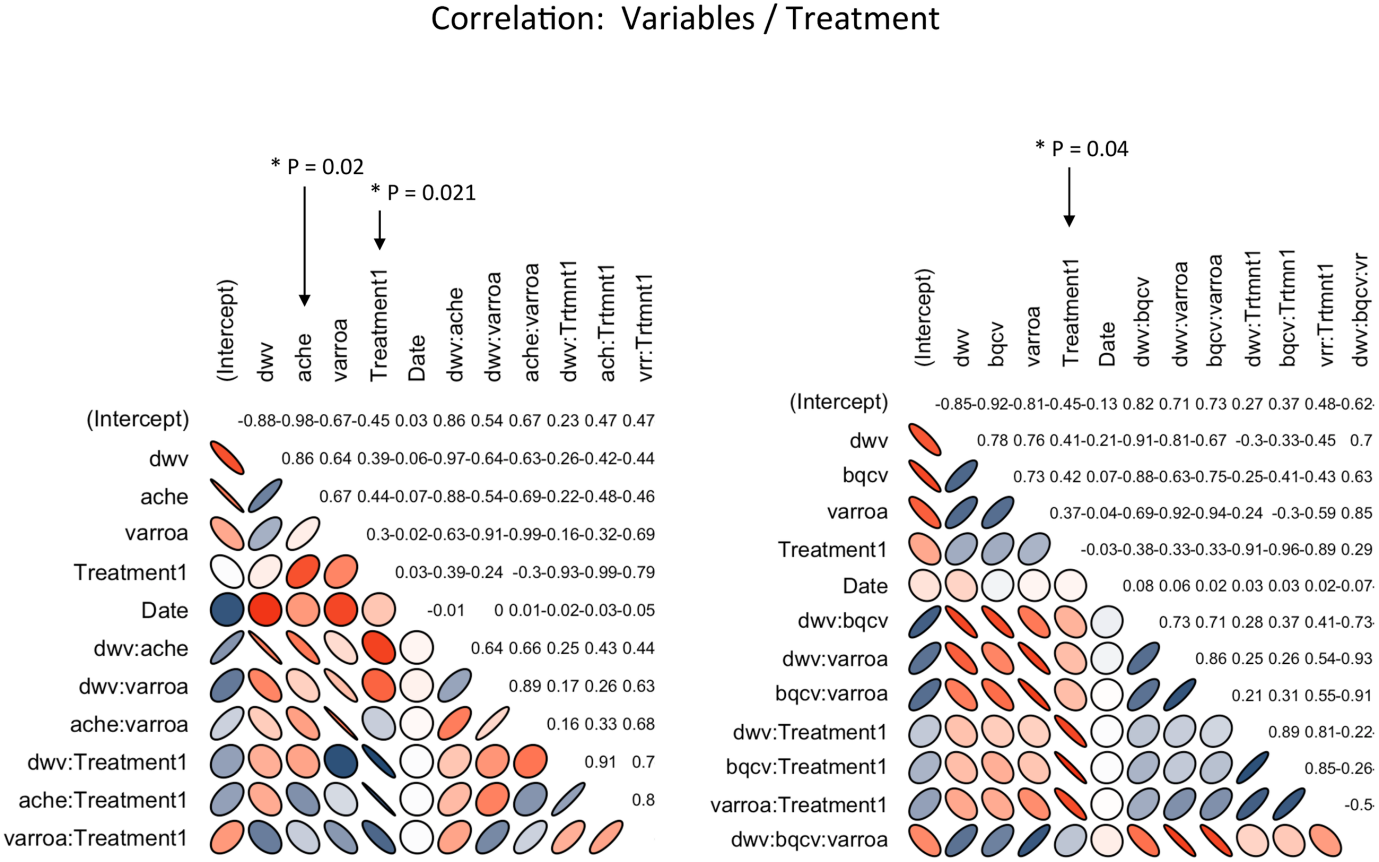
Overtime significant correlations between studied variables (AChE, DWV, BQCV and varroa) and the treatment factor. P values are * P < 0.05, r values are indicated for each pair of variable in the correlation matrixes and are calculated based on the linear mixed models of the fixed effect by allowing interaction between variables.

The treatment factor based on the design of our experiment reflects the variables’ contrast between 16 honeybee colonies placed in two distinct agricultural areas containing neonicotinoid treated cornfields and 16 others located in two organic cornfield areas. The correlation matrixes generated via the linear mixed models, on overtime-variable expressions, showed significant correlations among some variables and the treatment factor, Fig 6 and S2 Fig. As a pair-correlation, significant correlations (P = 0.02) were recorded between the treatment and AChE expression (r = 0.44) as well as DWV (r = 0.39) and varroa (r = 0.3), Fig 6. Beside that, a multiple correlation (P = 0.04) was detected for three pathogens (DWV, BQCV and varroa), these pathogens significantly correlated with the treatment factor (r = 0.29), Fig 6 and S2 Fig. No correlation was recorded between both brood and weight developments and the treatment factor. Only statistically significant correlations (P < 0.05) are shown and discussed in the text.

## Discussion

Over the last 20 years, neonicotinoids have emerged as the most widely used class of insecticide. Currently neonicotinoids are permitted in more than 120 countries, on more than 1000 different crops [60]. The evidence suggests that most neonicotinoids are highly persistent in water, soil and sediments [61]. Furthermore, neonicotinoids can also accumulate in soil after repeated use [8], which increases their absorption by subsequent cultivated crops or plants in the polluted soils. The study of pesticide toxicity to honeybees in the field presents significant challenges, especially when dealing with sublethal toxicity.

The four apiaries we studied in our experiment are located southwest of Quebec City, in an area dominated by corn cultivation (Fig 1). In recent years, different levels of honeybee mortality were reported by local beekeepers in the region. In the first year of our experiment, among the 32 studied colonies (16 colonies per treatment, Fig 1 and Table 1), 2 colonies perished in treated fields while one colony was lost in an untreated field. Although colony death could be related to numerous factors and not only to pesticide use, this remains an interesting observation. The cause of death differed among the three colonies. Those in the treated cornfields gradually perished after a remarkable decrease in the number of eggs laid by the queen, and symptoms of deformed wings were also recorded. Conversely, the colony located in untreated cornfields did not exhibit any disease symptoms and failed to re-queen and died at the very beginning of the experiment.

Comparisons of AChE activity over all colonies revealed no significant differences between honeybee colonies located in treated and untreated cornfields (Table 2, Fig 2a). Palynological analysis revealed that among the thirty-two studied colonies, five colonies had collected corn pollen (R2, R8, R26: untreated fields and R12, R24: treated fields). Although corn pollen was at lower concentration (1%) when compared to another recent study (ranging from 2.6% to 82.7%) [62], AChE expression was significantly higher in honeybee hives placed in two replicated treated cornfields (T = 2.62, P = 0.01) (Table 2, Fig 2b). This result suggests that significantly elevated AChE expression in the colonies located in the two treated cornfields—which occurred concomitantly with the flowering period (July and August), declining in October-2012 (Fig 2b)—is very likely to have a causal link to the presence of the corn pollen collected from the surrounding treated cornfields. Although a positive correlation was established (P = 0.02, r = 0.44) between the increase of AChE and the treatment factor (Fig 6 and S2 Fig), liquid chromatography-mass spectrometry (LC-MS) failed to detect any neonicotinoid in the analyzed pollen (Table 3). This result is not surprising because on one end, proportion of collected corn pollen by honeybee was only 1%, and on the other, measured concentrations of clothianidin in corn flowers of apiary 3 were low (3.7 ng/g). Although we can not exclude the probable contribution of other factors in the increase of AChE expression, these results are quite similar to those of [63] in which only trace (1 ng/g) of neonicotinoids resulting from insecticide seed treatments were identified in pollen collected by honeybees.

Among the three studied viruses, BQCV and DWV were identified at various levels in several hives. IAPV, on the other hand, was absent. Infection with BQCV was significantly higher in the hives located in treated cornfields right after the corn flowering period (Table 2, Fig 3). The varroa mite is a known vector of BQCV virus and other pathogens found in honeybees [64–67]. Our results showed significantly higher levels of varroa infection in colonies located in treated cornfields compared to those of the untreated fields (Table 2, Fig 4). Taken together, our data suggests that honeybee colonies placed next to neonicotinoid treated cornfields are subjected to a higher level of both viral replication and varroa mite load. Higher load of varroa mite could in turn favor viral transmission to honeybee [68]. The neonicotinoid triggering of viral pathogen replication was demonstrated to occur *in vitro* at sublethal doses [69]. Therefore, it remains possible that foraging in neonicotinoid treated cornfields impairs honeybee immunity and decreases their capacity to control the different hive’s pathogens. Chemical agents that promote honeybee susceptibility to pathogens have been demonstrated in previous studies, notably in a link between microsporidia (*Nosema* sp.) infection and neonicotinoid pesticides [70]. Furthermore in our study, genetic background—which could play a confounding role—can be largely discounted given that all colonies shared similar ancestry (Lineage C) and hives were randomly assigned between treatments. The latter parameter was taken into account in all our statistical analyses by considering the ‘apiary location’ as a random factor in the linear mixed models used. Interestingly, the statistical correlations conducted on our dataset link again the treatment factor in a positive correlation (P = 0.04, r = 0.29) with three different pathogens (DWV, BQCV and varroa infestation), Fig 6 and S2 Fig. Then this correlation suggests that proximity of bee colonies to treated cornfields lead to subtle increases of these pathogens. The significantly higher pathogens (BQCV and varroa infestation) as well as AChE expression in the colonies of the treated cornfields, evidenced in our study, can be a result of an indirect pesticide effect on honeybee health. Such observations may result from both an alteration of the bees’ hygienic system and immune response [71, 72].

Concerning the two biological traits (weight and brood) investigated in our study, they do not seem to be clearly or directly affected by the treatment factor *in vivo*. Colony weight gain (kg) revealed significant differences between hives located in the treated cornfields and untreated ones on two time points only (17 and 24-October, 2012) (Fig 5a). However, the brood development shows no significant differences between the two hives’ groups (treated and untreated), Fig 5b. Interestingly, the relatively better weight gain (17 and 24-October, 2012) of the treatment hives does not follow up with a better brooding (Fig 5a and 5b), which indicates that the mass gain is due to better honey and/or pollen collections, not necessarily to better colony size. In conclusion, it seems that neonicotinoid seed treatment had subtle impacts on honeybee brood and weight at the timescales addressed in our study. Similar data was documented on honeybees foraging in corn and canola fields treated with clothianidin [73].

## Conclusion

Our data showed that honeybee colonies placed in cornfields treated with neonicotinoid coated seeds experienced significantly higher varroa mite loads, and higher BQCV prevalence than colonies which were placed in control cornfields. Moreover, for colonies that had collected corn pollen, AChE levels were significantly higher in honeybees located in the treated cornfields than those of the untreated cornfields. Although AChE expression as well as BQCV, DWV and varroa infection were significantly correlated with the treatment factor, no neonicotinoids were detected in the bee hive products, but in the corn flowers. This suggests an indirect effect of the neonicotinoids on honeybee health in the fields. Therefore coupling more sensitive methods, such as polyclonal antibody-based enzyme-linked immunosorbent assay [74], with other biomarkers are strongly needed to provide rapid, efficient and cost effective tools for in-field monitoring.

## Supporting Information

S1 Fig Pollen grains. Diver pollen grain identified under the microscopy including grains of corn pollen *Z*. *mays*.
(PDF)

S2 Fig Correlation output. Linear mixed model output matrixes showing the different correlations between variables and the treatment factor.
(PDF)
