# Supplementary figures and images for: Neonicotinoid-Coated Zea mays Seeds Indirectly Affect Honeybee Performance and Pathogen Susceptibility in Field Trials
Source: PLoS One. 2015 May 18;10(5):e0125790. doi: 10.1371/journal.pone.0125790 (PMC4436261; doi:10.1371/journal.pone.0125790)

DSCN7419

R24 02/08/12

120µm

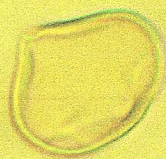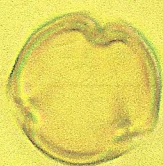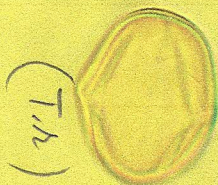

(T.h)

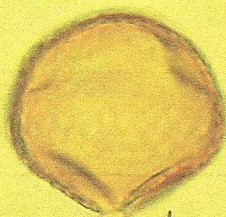

Trifolium type praefense  
(T.p)

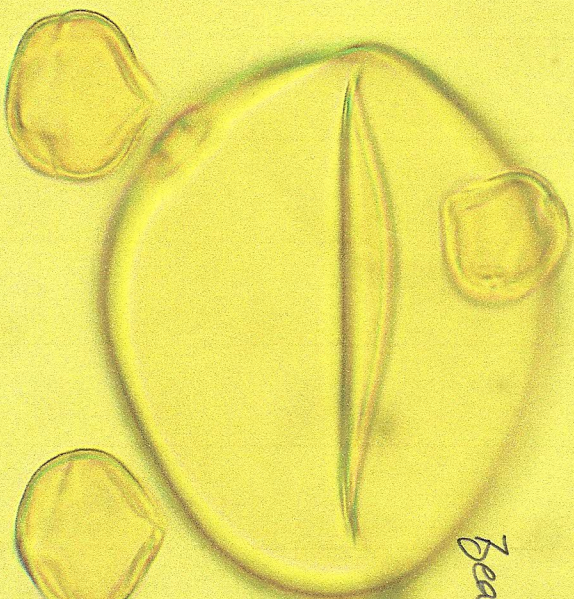

Bea

T.n

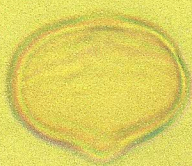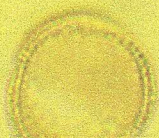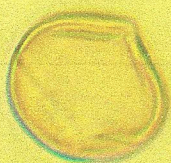

Supplement: S1 Fig — Diver pollen grain identified under the microscopy including grains of corn pollen Z. mays. (PDF) [file pone.0125790.s001.pdf]
