# Supplementary material for: Neonicotinoid-Coated Zea mays Seeds Indirectly Affect Honeybee Performance and Pathogen Susceptibility in Field Trials
Source: PLoS One. 2015 May 18;10(5):e0125790. doi: 10.1371/journal.pone.0125790 (PMC4436261; doi:10.1371/journal.pone.0125790)
Supplement: S2 Fig — Linear mixed model output matrixes showing the different correlations between variables and the treatment factor. (PDF) [file pone.0125790.s002.pdf]

Date as fixed effect, Apiary as random, Allowing interaction between variables

```
#########################################################################################
BQCV
##########################################################################################

Linear mixed model fit by maximum likelihood  ['merModLmerTest']
Formula: databees$bqcv ~ dwv * ache * varroa * Treatment + Date + (1 |      Apiary)
   Data: databees

     AIC      BIC   logLik deviance df.resid
   483.3    537.0   -222.6    445.3      106

Scaled residuals:
    Min      1Q  Median      3Q     Max
-2.1310 -0.7154 -0.2174  0.6100  2.6731

Random effects:
 Groups   Name        Variance Std.Dev.
 Apiary   (Intercept) 0.0153   0.1237
 Residual             2.0491   1.4315
Number of obs: 125, groups: Apiary, 4

Fixed effects:
                          Estimate Std. Error       df t value Pr(>|t|)
(Intercept)                12.9012     3.6948 121.2800   3.492  0.00067 ***
dwv                        -0.2927     0.6913 120.2100  -0.423  0.67276
ache                       -4.8183     2.1333 121.0500  -2.259  0.02570 *
varroa                     -5.4298     5.4733 119.5000  -0.992  0.32318
Treatment                 -19.1765     8.2633 121.1200  -2.321  0.02198 *
Date                       -0.5633     0.1509 121.5600  -3.732  0.00029 ***
dwv:ache                    0.2908     0.4000 119.2400   0.727  0.46861
dwv:varroa                  0.4005     1.2170 119.6500   0.329  0.74263
ache:varroa                 3.4787     3.1361 119.6000   1.109  0.26954
dwv:Treatment               4.1424     2.6624 122.3100   1.556  0.12231
ache:Treatment             10.6525     4.4005 120.7500   2.421  0.01698 *
varroa:Treatment           12.2821     7.8996 119.2400   1.555  0.12265
dwv:ache:varroa            -0.3121     0.6933 119.7100  -0.450  0.65345
dwv:ache:Treatment         -2.1893     1.3703 121.7900  -1.598  0.11271
dwv:varroa:Treatment       -2.0881     2.1793 119.7100  -0.958  0.33990
ache:varroa:Treatment      -6.8555     4.3559 119.3200  -1.574  0.11817
dwv:ache:varroa:Treatment   1.2724     1.1757 119.6300   1.082  0.28132
---
Signif. codes:  0 ‘***’ 0.001 ‘**’ 0.01 ‘*’ 0.05 ‘.’ 0.1 ‘ ’ 1

Correlation of Fixed Effects:
            (Intr) dwv    ache   varroa Trtmnt Date   dwv:ch dwv:vr ach:vr dwv:Tr ach:Tr
vrr:Tr dwv:c: dwv:c:T dwv:v:T ach::T
dwv         -0.878
ache        -0.982  0.865
varroa      -0.675  0.637  0.670
Treatment   -0.446  0.390  0.436  0.301
Date         0.031 -0.063 -0.069 -0.017  0.029
dwv:ache     0.861 -0.975 -0.880 -0.635 -0.385 -0.006
```

```
dwv:varroa   0.538 -0.643 -0.537 -0.906 -0.240  0.004  0.641
ache:varroa  0.669 -0.633 -0.689 -0.988 -0.299  0.011  0.656  0.894
dwv:Tretmnt  0.227 -0.257 -0.222 -0.165 -0.934 -0.025  0.253  0.167  0.164
ache:Trtmnt  0.474 -0.415 -0.480 -0.324 -0.991 -0.031  0.427  0.260  0.333  0.912
varr:Trtmnt  0.466 -0.438 -0.461 -0.692 -0.795 -0.046  0.440  0.628  0.684  0.701  0.796
dwv:ach:vrr -0.538  0.641  0.558  0.899  0.240 -0.008 -0.666 -0.987 -0.910 -0.166 -0.270
-0.623
dwv:ch:Trtm -0.251  0.283  0.255  0.185  0.935  0.024 -0.292 -0.187 -0.191 -0.994 -0.925
-0.710  0.194
dwv:vrr:Trt -0.299  0.356  0.297  0.505  0.811  0.035 -0.358 -0.558 -0.499 -0.846 -0.802
-0.896  0.551  0.854
ach:vrr:Trt -0.481  0.453  0.493  0.710  0.771  0.036 -0.472 -0.644 -0.719 -0.671 -0.789
-0.989  0.655  0.692   0.878
dwv:ch:vr:T  0.316 -0.376 -0.327 -0.530 -0.788 -0.024  0.393  0.582  0.536  0.818  0.792
0.893 -0.589 -0.837  -0.991  -0.892


###########################################################################################
DWV
###########################################################################################
Linear mixed model fit by maximum likelihood  ['merModLmerTest']
Formula: databees$dwv ~ bqcv * ache * varroa * Treatment + Date + (1 |      Apiary)
   Data: databees

     AIC      BIC   logLik deviance df.resid
   500.7    554.4   -231.3    462.7      106

Scaled residuals:
    Min      1Q  Median      3Q     Max
-2.1855 -0.7253 -0.1208  0.7115  2.2402

Random effects:
 Groups   Name        Variance Std.Dev.
 Apiary   (Intercept) 0.07284  0.2699
 Residual             2.31990  1.5231
Number of obs: 125, groups: Apiary, 4

Fixed effects:
                            Estimate Std. Error        df t value Pr(>|t|)
(Intercept)                 -0.71726    5.69049 121.50000  -0.126   0.8999
bqcv                         0.31095    0.83059 120.16000   0.374   0.7088
ache                         0.29021    3.11686 121.77000   0.093   0.9260
varroa                       6.79138    7.07281 120.54000   0.960   0.3389
Treatment                   11.44966   10.33849 120.82000   1.107   0.2703
Date                         1.13336    0.14369 123.82000   7.888 1.38e-12 ***
bqcv:ache                    0.05302    0.49420 120.82000   0.107   0.9147
bqcv:varroa                 -1.63238    1.36800 120.52000  -1.193   0.2351
ache:varroa                 -3.01242    3.79833 120.40000  -0.793   0.4293
bqcv:Treatment              -2.26555    1.96521 120.06000  -1.153   0.2513
ache:Treatment              -6.02029    5.73336 120.42000  -1.050   0.2958
varroa:Treatment           -21.62695   11.76479 121.68000  -1.838   0.0685 .
bqcv:ache:varroa             0.65620    0.75553 120.43000   0.869   0.3868
bqcv:ache:Treatment          1.10634    1.13215 120.08000   0.977   0.3304
bqcv:varroa:Treatment        4.32992    2.30035 121.03000   1.882   0.0622 .
```

```
ache:varroa:Treatment       10.26471    6.42239 121.13000   1.598   0.1126
bqcv:ache:varroa:Treatment  -2.03966    1.29783 120.65000  -1.572   0.1187
---
Signif. codes:  0 ‘***’ 0.001 ‘**’ 0.01 ‘*’ 0.05 ‘.’ 0.1 ‘ ’ 1

Correlation of Fixed Effects:
            (Intr) bqcv   ache   varroa Trtmnt Date   bqcv:c bqcv:v ach:vr bqcv:T ach:Tr
vrr:Tr bqcv:: bqcv:c:T bqcv:v:T ach::T
bqcv        -0.925
ache        -0.980  0.938
varroa      -0.770  0.705  0.749
Treatment   -0.552  0.507  0.535  0.428
Date        -0.024 -0.033 -0.075  0.070  0.081
bqcv:ache    0.843 -0.964 -0.902 -0.636 -0.457  0.099
bqcv:varroa  0.561 -0.582 -0.554 -0.902 -0.314 -0.078  0.547
ache:varroa  0.774 -0.729 -0.776 -0.987 -0.430 -0.060  0.691  0.898
bqcv:Trtmnt  0.392 -0.420 -0.391 -0.303 -0.947 -0.054  0.401  0.251  0.312
ache:Trtmnt  0.536 -0.506 -0.535 -0.415 -0.987 -0.070  0.479  0.310  0.429  0.948
varr:Trtmnt  0.463 -0.424 -0.451 -0.601 -0.815 -0.034  0.383  0.542  0.593  0.756  0.807
bqcv:ch:vrr -0.553  0.605  0.574  0.867  0.308  0.055 -0.611 -0.980 -0.893 -0.260 -0.318
-0.521
bqcv:ch:Trt -0.370  0.418  0.388  0.283  0.913  0.037 -0.429 -0.245 -0.306 -0.982 -0.943
-0.735  0.271
bqcv:vrr:Tr -0.333  0.347  0.331  0.535  0.745  0.026 -0.327 -0.593 -0.533 -0.767 -0.749
-0.940  0.582  0.761
ach:vrr:Trt -0.458  0.431  0.460  0.583  0.816  0.031 -0.409 -0.531 -0.591 -0.768 -0.828
-0.989  0.528  0.769    0.940
bqcv:ch:v:T  0.322 -0.353 -0.335 -0.504 -0.733 -0.020  0.357  0.570  0.519  0.770  0.759
0.913 -0.582 -0.791   -0.986   -0.936



###########################################################################################
ACHE
###########################################################################################


Linear mixed model fit by maximum likelihood  ['merModLmerTest']
Formula: databees$ache ~ dwv * bqcv * varroa * Treatment + Date + (1 |      Apiary)
   Data: databees

     AIC      BIC   logLik deviance df.resid
    44.3     98.0     -3.1      6.3      106

Scaled residuals:
    Min      1Q  Median      3Q     Max
-4.1343 -0.4840  0.1183  0.6344  2.0283

Random effects:
 Groups   Name        Variance Std.Dev.
 Apiary   (Intercept) 0.00000  0.0000
 Residual             0.06156  0.2481

Number of obs: 125, groups: Apiary, 4
```

```
Fixed effects:
                            Estimate Std. Error        df t value Pr(>|t|)    
(Intercept)                 2.161275   0.308206 124.950000   7.012  1.3e-10 ***
dwv                        -0.049698   0.064380 124.950000  -0.772   0.4416    
bqcv                       -0.107375   0.061701 124.950000  -1.740   0.0843 .  
varroa                     -0.388218   0.430370 124.950000  -0.902   0.3688    
Treatment                   0.446184   0.672911 124.950000   0.663   0.5085    
Date                        0.061029   0.027138 124.950000   2.249   0.0263 *  
dwv:bqcv                    0.005855   0.012289 124.950000   0.476   0.6346    
dwv:varroa                  0.052630   0.094181 124.950000   0.559   0.5773    
bqcv:varroa                 0.084171   0.088936 124.950000   0.946   0.3458    
dwv:Treatment              -0.302981   0.206077 124.950000  -1.470   0.1440    
bqcv:Treatment             -0.167538   0.150699 124.950000  -1.112   0.2684    
varroa:Treatment           -0.849082   0.726835 124.950000  -1.168   0.2450    
dwv:bqcv:varroa            -0.009517   0.019905 124.950000  -0.478   0.6334    
dwv:bqcv:Treatment          0.079849   0.043938 124.950000   1.817   0.0716 .  
dwv:varroa:Treatment        0.393552   0.213210 124.950000   1.846   0.0673 .  
bqcv:varroa:Treatment       0.224105   0.158859 124.950000   1.411   0.1608    
dwv:bqcv:varroa:Treatment  -0.086501   0.043544 124.950000  -1.987   0.0492 *  
---
Signif. codes:  0 ‘***’ 0.001 ‘**’ 0.01 ‘*’ 0.05 ‘.’ 0.1 ‘ ’ 1

Correlation of Fixed Effects:
            (Intr) dwv    bqcv   varroa Trtmnt Date   dwv:bq dwv:vr bqcv:v dwv:Tr bqcv:T
vrr:Tr dwv:b: dwv:b:T dwv:v:T bqc::T
dwv         -0.847                                                                      
bqcv        -0.919  0.780                                                               
varroa      -0.805  0.757  0.726                                                        
Treatment   -0.446  0.406  0.415  0.372                                                 
Date        -0.133 -0.211  0.067 -0.036 -0.027                                          
dwv:bqcv     0.817 -0.908 -0.884 -0.693 -0.382  0.084                                   
dwv:varroa   0.711 -0.807 -0.631 -0.918 -0.331  0.061  0.729                            
bqcv:varroa  0.727 -0.672 -0.754 -0.937 -0.334  0.018  0.707  0.863                     
dwv:Tretmnt  0.269 -0.305 -0.246 -0.235 -0.914  0.031  0.281  0.250  0.209              
bqcv:Trtmnt  0.369 -0.331 -0.406 -0.299 -0.964  0.030  0.367  0.262  0.310  0.888       
varr:Trtmnt  0.477 -0.447 -0.430 -0.592 -0.889  0.017  0.410  0.543  0.555  0.808  0.851
dwv:bqcv:vr -0.617  0.703  0.629  0.845  0.289 -0.071 -0.729 -0.932 -0.906 -0.217 -0.262
-0.500
dwv:bqcv:Tr -0.226  0.258  0.246  0.194  0.896 -0.041 -0.281 -0.205 -0.198 -0.976 -0.919
-0.781  0.205
dwv:vrr:Trt -0.313  0.357  0.278  0.406  0.849 -0.030 -0.322 -0.442 -0.381 -0.917 -0.819
-0.913  0.412  0.881
bqcv:vrr:Tr -0.403  0.382  0.420  0.526  0.865 -0.040 -0.398 -0.485 -0.560 -0.788 -0.887
-0.963  0.509  0.803   0.882
dwv:bqcv::T  0.279 -0.326 -0.286 -0.387 -0.842  0.051  0.335  0.427  0.414  0.900  0.855
0.898 -0.458 -0.907  -0.971  -0.922
```
